# Twenty Years On: RECIST as a Biomarker of Response in Solid Tumours an EORTC Imaging Group – ESOI Joint Paper

**DOI:** 10.3389/fonc.2021.800547

**Published:** 2022-01-10

**Authors:** Laure Fournier, Lioe-Fee de Geus-Oei, Daniele Regge, Daniela-Elena Oprea-Lager, Melvin D’Anastasi, Luc Bidaut, Tobias Bäuerle, Egesta Lopci, Giovanni Cappello, Frederic Lecouvet, Marius Mayerhoefer, Wolfgang G. Kunz, Joost J. C. Verhoeff, Damiano Caruso, Marion Smits, Ralf-Thorsten Hoffmann, Sofia Gourtsoyianni, Regina Beets-Tan, Emanuele Neri, Nandita M. deSouza, Christophe M. Deroose, Caroline Caramella

**Affiliations:** ^1^ Imaging Group, European Organisation of Research and Treatment in Cancer (EORTC), Brussels, Belgium; ^2^ European Society of Oncologic Imaging (ESOI), European Society of Radiology, Vienna, Austria; ^3^ Université de Paris, Assistance Publique–Hôpitaux de Paris (AP-HP), Hopital europeen Georges Pompidou, Department of Radiology, Paris Cardiovascular Research Center (PARCC) Unité Mixte de Recherche (UMRS) 970, Institut national de la santé et de la recherche médicale (INSERM), Paris, France; ^4^ Department of Radiology, Leiden University Medical Center, Leiden, Netherlands; ^5^ Biomedical Photonic Imaging Group, University of Twente, Enschede, Netherlands; ^6^ Department of Surgical Sciences, University of Turin, Turin, Italy; ^7^ Radiology Unit, Candiolo Cancer Institute, Fondazione del Piemonte per l’Oncologia-Istituto Di Ricovero e Cura a Carattere Scientifico (FPO-IRCCS), Turin, Italy; ^8^ Department of Radiology & Nuclear Medicine, Cancer Centre Amsterdam, Amsterdam University Medical Centers [Vrije Universiteit (VU) University], Amsterdam, Netherlands; ^9^ Medical Imaging Department, Mater Dei Hospital, University of Malta, Msida, Malta; ^10^ College of Science, University of Lincoln, Lincoln, United Kingdom; ^11^ Institute of Radiology, University Hospital Erlangen, Friedrich-Alexander-Universität Erlangen-Nürnberg (FAU), Erlangen, Germany; ^12^ Nuclear Medicine Unit, Istituto Di Ricovero e Cura a Carattere Scientifico (IRCCS) – Humanitas Research Hospital, Milan, Italy; ^13^ Department of Radiology, Institut de Recherche Expérimentale et Clinique (IREC), Cliniques Universitaires Saint Luc, Université Catholique de Louvain (UCLouvain), Brussels, Belgium; ^14^ Department of Radiology, Memorial Sloan Kettering Cancer Center, New York, NY, United States; ^15^ Department of Biomedical Imaging and Image-guided Therapy, Medical University of Vienna, Vienna, Austria; ^16^ Department of Radiology, University Hospital, Ludwig Maximilian University (LMU) Munich, Munich, Germany; ^17^ Department of Radiotherapy, University Medical Center Utrecht, Utrecht University, Utrecht, Netherlands; ^18^ Department of Medical-Surgical Sciences and Translational Medicine, Sapienza University of Rome, Rome, Italy; ^19^ Department of Radiology & Nuclear Medicine, Erasmus MC, University Medical Centre Rotterdam, Rotterdam, Netherlands; ^20^ Brain Tumour Centre, Erasmus Medical Centre (MC) Cancer Institute, Rotterdam, Netherlands; ^21^ Institute and Policlinic for Diagnostic and Interventional Radiology, University Hospital, Carl-Gustav-Carus Technical University Dresden, Dresden, Germany; ^22^ Department of Radiology, School of Medicine, National and Kapodistrian University of Athens, Areteion Hospital, Athens, Greece; ^23^ Department of Radiology, The Netherlands Cancer Institute, Amsterdam, Netherlands; ^24^ School For Oncology and Developmental Biology (GROW) School for Oncology and Developmental Biology, Maastricht University, Maastricht, Netherlands; ^25^ Diagnostic and Interventional Radiology, Department of Translational Research and of New Surgical and Medical Technologies, University of Pisa, Pisa, Italy; ^26^ Division of Radiotherapy and Imaging, The Institute of Cancer Research and Royal Marsden National Health Service (NHS) Foundation Trust, London, United Kingdom; ^27^ European Imaging Biomarkers Alliance (EIBALL), European Society of Radiology, Vienna, Austria; ^28^ Quantitative Imaging Biomarkers Alliance, Radiological Society of North America, Oak Brook, IL, United States; ^29^ Nuclear Medicine, University Hospitals Leuven, Leuven, Belgium; ^30^ Nuclear Medicine & Molecular Imaging, Department of Imaging and Pathology, Katholieke Universiteit (KU) Leuven, Leuven, Belgium; ^31^ Radiology Department, Hôpital Marie Lannelongue, Groupe Hospitalier Paris Saint Joseph Centre International des Cancers Thoraciques, Université Paris-Saclay, Le Plessis-Robinson, France

**Keywords:** tumour, biomarker, imaging, response, RECIST

## Abstract

Response evaluation criteria in solid tumours (RECIST) v1.1 are currently the reference standard for evaluating efficacy of therapies in patients with solid tumours who are included in clinical trials, and they are widely used and accepted by regulatory agencies. This expert statement discusses the principles underlying RECIST, as well as their reproducibility and limitations. While the RECIST framework may not be perfect, the scientific bases for the anticancer drugs that have been approved using a RECIST-based surrogate endpoint remain valid. Importantly, changes in measurement have to meet thresholds defined by RECIST for response classification within thus partly circumventing the problems of measurement variability. The RECIST framework also applies to clinical patients in individual settings even though the relationship between tumour size changes and outcome from cohort studies is not necessarily translatable to individual cases. As reproducibility of RECIST measurements is impacted by reader experience, choice of target lesions and detection/interpretation of new lesions, it can result in patients changing response categories when measurements are near threshold values or if new lesions are missed or incorrectly interpreted. There are several situations where RECIST will fail to evaluate treatment-induced changes correctly; knowledge and understanding of these is crucial for correct interpretation. Also, some patterns of response/progression cannot be correctly documented by RECIST, particularly in relation to organ-site (e.g. bone without associated soft-tissue lesion) and treatment type (e.g. focal therapies). These require specialist reader experience and communication with oncologists to determine the actual impact of the therapy and best evaluation strategy. In such situations, alternative imaging markers for tumour response may be used but the sources of variability of individual imaging techniques need to be known and accounted for. Communication between imaging experts and oncologists regarding the level of confidence in a biomarker is essential for the correct interpretation of a biomarker and its application to clinical decision-making. Though measurement automation is desirable and potentially reduces the variability of results, associated technical difficulties must be overcome, and human adjudications may be required.

## Introduction

Imaging plays a major role in the evaluation of tumour response to cancer treatments. It provides an objective *in-vivo* measurement of tumour burden, and helps oncologists determine whether a treatment should be pursued, interrupted or adapted.

Response evaluation criteria in solid tumours (RECIST) v1.1 currently is the reference standard for evaluating efficacy of therapies in patients with solid tumours who are included in clinical trials, and it is widely used and accepted by regulatory agencies (1). However, many publications question both the reproducibility and the clinical relevance of RECIST. This paper is an expert statement aiming to answer some of the questions regarding the principles underlying RECIST and its reproducibility compared to other biomarkers, as well as the limitations to its application and continued role in an era where other biomarkers exist that are more explicitly geared towards tumour-specific properties.

## How Were RECIST Thresholds Established?

RECIST has instituted several overarching principles underpinning its approach to tumour response evaluation. Primarily, RECIST defines which lesions are measurable in a reliable manner. Among these, it defines a maximal number of lesions (‘target lesions’) to be measured to yield a quantitative value representative of tumour burden. The remainder are considered ‘non-target lesions’ and are evaluated qualitatively. On follow-up scans, new lesions indicate progression (**Table 1**). The threshold for response is defined as a decrease of at least 30% of sum of diameters (SOD) of target lesions compared to baseline, AND no progression of non-target lesions AND no new lesions. The threshold for progressive disease (PD) is defined as an increase of at least 20% of SOD of target lesions compared to nadir AND/OR unequivocal progression of non-target lesions AND/OR appearance of new lesions.

**Table 1:** RECIST categories of response.

Overall Response	Target Lesions	Non Target Lesions	New Lesions
**Definition**	•Lesions with longestdiameter≥10 mm and limits that are sufficiently well defined for their measurement to be considered reliable •Lymph nodes: measurement of short axis, target lesion if short-axis measures≥15 mm • Maximum number of selected target lesions 5/patient and 2/organ	•Lesions that are too small (< 10 mm) •Lesions for which measurement is considered unreliable as their limits are difficult to define (bone or leptomeningeal lesions, ascites, pleural or pericardial effusion, lymphangitic carcinomatosis etc.) •Measurable lesions not selected as target lesions •Lymph nodes: measurement of short axis, non-target lesion if 10 mm ≤ short-axis diameter < 15 mm •Levels of tumour markers > normal (if relevant and predefined)	
**Complete response (CR)**	• Disappearance of all target lesions and all nodes have short axis < 10 mm	• Disappearance of all non-target lesions and normalisation of tumour marker levels	• No
**Partial response (PR)**	•≥ 30% decrease in the sum of target lesions taking as reference the baseline sum	•No progression	• No
**Stable disease (SD)**	•Neither response nor progression	• Persistence of one or more non-target lesions and/or tumour marker levels > normal	• No
**Progressive disease (PD):** response is PD if at least one category of lesions meets progression criteria	•≥ 20% increase in the sum of target lesions taking as reference the smallest sum measured during follow-up (nadir) and ≥ 5 mm in absolute value	• ‘Unequivocal’ progression (assessed qualitatively) in lesion size (an increase in size of a single lesion is not sufficient)	• Yes [appearance of new unequivocally metastatic lesion(s)]

The first publication addressing thresholds for determining treatment efficacy was published by Moertel and Hanley in 1976 (2). In this study, 16 observers were asked to measure by clinical examination using a calliper the diameters of solid spheres of variable sizes arranged randomly underneath a mattress. Authors suggested the product of two diameters should be used, as this would be more reliable if lesions were not spherical. For this ‘clinical’ estimate, a 50% reduction in the product of two diameters was shown to have an acceptable measurement error estimated between 7-8%. Interestingly, the authors specifically stated that “the purpose was not to predict long-term efficacy but to determine what change in bidimensional size could be confidently considered a change”. Progression, on the other hand, was defined as an increase in the product of diameters ≥ 25%, but the authors could not justify this cut-off, other than by specifying it “should not necessarily be regarded as influencing the management of the patient”.

In 1979, the World Health Organization (WHO) provided recommendations for the evaluation of cancer treatments in clinical trials on imaging. Criteria were based not only on the bidimensional measurement of lesions on clinical examination, but also CT or standard radiography (3), transposing results of Moertel and Hanley’s study and setting cut-offs for definition of response to -50% and of progression to +25%. However, many technical aspects were not detailed, such as the number of lesions to be measured or what constituted a measurable lesion.

In 2000, a working group of European, American and Canadian cancer research organizations (EORTC, NCI, NCIC) defined the Response Evaluation Criteria In Solid Tumours – RECIST (4). They used data from over 4,600 patients enrolled in 14 clinical trials to formulate criteria based on imaging. RECIST used unidimensional measurement of lesions, justified by an extensive comparison of methods of measurement (1D *vs*. 2D) (5). Moreover, this working group specified conditions of measurement, number of lesions, and detailed how to document progression. Regarding cut-off values for response and progression, the -50% value for response for bidimensional measurement was altered to -30% for unidimensional measurements, and the +25% value for progression for bidimensional measurement was altered to +20% for unidimensional measurements (**Table 2**).

**Table 2:** Relationship between diameter and corresponding volume.

Diameter (“long axis”)	Percentage of variation	Corresponding volume	Percentage of variation
20 mm		4.2 cm3	
26 mm	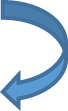 +30%	9.2 cm3	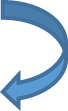 +120%
34 mm	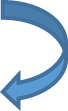 +30%	20.6 cm3	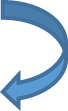 + 120%
27 mm	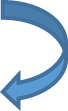 -20%	10.3 cm3	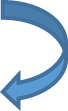 -50%

Repeated measurements are given for a theoretical lesion including diameter measured in a single dimension (long axis), percent changes between measurements, and the corresponding volume assuming the lesion is a sphere and percent changes in volume.

RECIST was then revised in 2009 (version 1.1) (1), introducing specific rules for measurement of small axis of lymph nodes and reducing the number of target lesions to five per patient. This new version was also based on data analysis, including a literature review and a simulation using a database of over 6,500 patients and 18,000 lesions. The number of target lesions for example, was chosen by determining the minimum number for which response rates and time to progression were not altered from RECIST 1.0 results (6, 7).

### Statement #1

*RECIST thresholds were chosen to produce a comparable classification of patients in a given category of response when comparing trials or even when comparing patients, taking into account tumour measurement variability.*

## Do RECIST Categories Predict Outcome?

RECIST criteria were originally tested and validated to provide an objective and reproducible assessment of treatment effect in cancer patients, without any references to patient outcome (8). Yet it seems intuitive that when a tumour decreases in size, a patient will have a better outcome, and vice versa. There is evidence to support this, including some large studies, which pool data from various trials. In over 500 patients with metastatic colorectal cancer treated with combination chemotherapy, a decrease in size resulted in a decreased hazard ratio for overall survival (OS) (9). In a meta-analysis of 24 phase I trials, a linear relationship was shown between change in tumour size and survival (10). In a pooled analysis of over 2,700 patients with metastatic renal cell carcinoma treated with anti-angiogenic agents, tumour shrinkage of ≥ 30% resulted in improved OS and progression-free survival (PFS) (11). In addition, the authors demonstrated that tumour shrinkage between 60% and 100% at 6-month follow-up represented an independent prognostic factor for OS. Litière et al. also demonstrated in an even larger pooled analysis of over 23,000 patients treated with targeted agents, chemotherapy or a combination thereof (12), that a decrease in tumour size was consistently associated with a lower hazard ratio, while an increase in size was associated with a higher hazard ratio.

Tumour response according to RECIST can only be quantified by a decrease in size or number of target lesions, as non-target lesions are not taken into consideration for partial response (PR). Regarding progression however, it is important to consider non-target lesions, as unequivocal progression of non-target lesions or emergence of new lesions defines tumour progression. In over 3,700 patients from 13 trials in the RECIST trial database, the presence of new lesions and progression of non-target lesions were most strongly associated with worse OS (hazard ratios range 1.5–2.3) regardless of tumour type, whereas percentage tumour growth in target lesions contributed less in a multivariate model of OS (13).

Finally, in two separate studies (14, 15), An et al. compared the predictive ability of RECIST categories vs. longitudinal tumour measurement–based continuous metrics and alternative categorical response metrics such as slope (absolute change in tumour size) and percent change (relative change in tumour size) to predict OS. The databases consisted respectively of almost 2,100 patients from 13 trials and over 1,500 patients from 3 trials with breast cancer, non–small cell lung cancer (NSCLC) or colorectal cancer. Although there seemed to be a slightly better performance for continuous variables, it was not statistically significant, which led the authors to conclude there was no evidence that growth rate or a continuous evaluation of percent change would improve prediction of outcome. However, it may be noted that timing of evaluations, particularly when considering non-continuous variables, may have an impact on their performance and results.

### Statement #2

*Tumour size changes correlate to outcome at a statistical (cohort) level.*

## How Reproducible Is RECIST?

When considering whether RECIST evaluates tumour response correctly, metrology principles guide us to consider two aspects (16): is the measurement “true” (when compared to a “real” value, which defines its accuracy), and is the measurement “precise” (i.e. repeatable and reproducible)?

Assessing accuracy of change in size measurements would require obtaining “true” values of change in size. As it is not possible to surgically excise all tumours for comparison with imaging, and often inaccurate to compare *ex vivo* with *in vivo* measurements, the true value of an imaging biomarker must be derived from data obtained through a combination of primary tumour excision and phantom studies.

**Figure 1:**
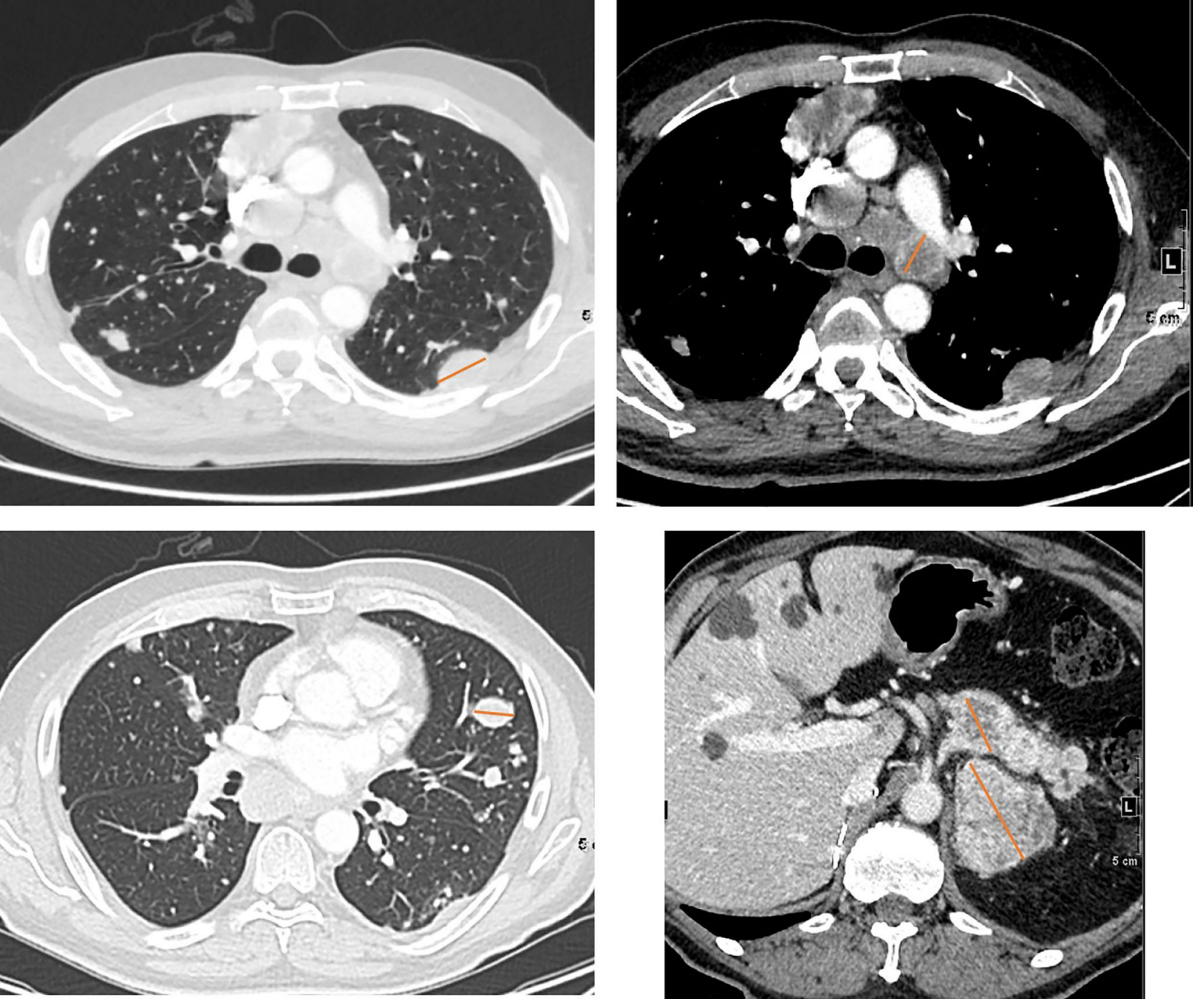
Selecting target lesions in a 58 yo patient with metastatic renal cell carcinoma. Multiple lung, lymph node, pancreatic and adrenal metastases are present. Lymph nodes should be sampled from different locations where possible. Selection of target lesions at baseline from multiple organ sites is important for response evaluation at a patient level.

Precision refers to the variability of the measurement process and can be evaluated by repeatability (when measurement conditions do not change) and reproducibility (when measurement conditions vary). The precision of RECIST and of response categories has been studied extensively. **Table 3** lists the documented reproducibility of RECIST and factors that may impact it. Overall, SOD reproducibility is in the order of +/-20% in multi-observer studies, and +/-10% in single observer studies (17). Important factors associated with RECIST measurement reproducibility are the choice and number of target lesions (**Figure 1**) and the experience of the reader(s). Where multiple target lesions are used, their selection affects variability: agreement ranges from 0.58 when different targets are chosen to 0.97 when the same targets are used (23). Variability also increases with the number of target lesions selected. For this reason, it has been recommended that a central review in clinical trials should include two readers and one adjudicator (29). Finally, reader experience has major impact on variability, from the selection of the correct reference examination (baseline vs. previous CT) to the detection and proper interpretation of new lesions (20, 21, 25, 26). Measurements of well-demarcated lesions and bigger lesions are also more reproducible (17–19), which vindicates RECIST recommendations for the choice of target lesions.

**Table 3:** RECIST reproducibility and factors impacting it.

Biomarker	Reproducibility			Factors impacting reproducibility
	95% limits of agreement	Kappa	Other	
RECIST (measurement) CT (size)	Per lesion - Intra-obs: -18% to 16% - Inter-obs: -22% to 25% (1 (17) Per sum of diameters - Intra-obs: -10% to 13% - Inter-obs: -20% to 20% Interval change in tumour burden (% change between time points) - -31% to 30% Repeatability (same image on repeat CT taken within 15 minutes) - -4% to +4% (18)	With target lesion selection - Intra-obs: 0.957 (19) - Inter-obs 0.954 (19) Target response classification - Inter-obs: 0.48 (20) to 0.66 (21) Non-target response classification - Inter-obs: 0.58 (20)	Lesion size ICC (22) -Pre-treatment: 0.72 -Post-treatment: 0.85 -Interval change: 0.70	-Selection of target lesions differs in 21 to 33% (17, 23, 24) -Practical training (ref 40)/expertise (21) -Same observer (17, 20) -Well delineated lesions (17, 19) -Lesions size (greater variability for smaller lesions) (18, 19) -Adjudication could reduce easily avoidable inconsistencies (20, 25)
RECIST (overall response)		With target lesion selection - Inter-obs: 0.97 (23) Without target lesion selection - Inter-obs: 0.51 (20), 0.53 (24, 26) to 0.58 (23)	-30% of patients classified differently in a cohort of 39 pts with 2 readers (26)	-Arbitrary nature of CR/PR/SD/PD categories (10) -Inconsistencies mainly due to interpretation of new lesions (20, 26) -Choice of target lesions
3D measurement	- Intra-obs: 0.4 to 33% according to automated volume measurement method (27)	Whole body volumetry - Inter-obs: 0.95 30)	-Discordant classification in overall response in 10 to 21% of patients according to automated volume measurement method (27)	-Time consuming (28) -Do not resolve the discrepancies linked to the choice of target lesions (24)

95% limits of agreement are derived from the Bland-Altman method comparing two measurements of the same variable. Kappa coefficients measure agreement between qualitative observations. ICC measures the reliability of measurements by comparing the variability of different ratings of the same subject to the total variation across all ratings and all subjects.

Intra-obs, intra-observer; Inter-obs, inter-observer; ICC, Intra-class coefficient; CR, complete response; PR, partial response; SD, stable disease; PD, progressive disease.

### Statement #3

*RECIST reproducibility is impacted by reader experience, choice of target lesions, lesion characteristics, and detection/interpretation of new lesions. At an individual level, this can result in patients being categorised incorrectly when values of SOD are near thresholds or when new lesions are either missed or incorrectly interpreted.*

## How Reproducible Are Other Biomarkers?

**Table 4** summarises repeatability and reproducibility of some of the other biomarkers suggested or used as alternatives to RECIST for evaluating response. With the abundance of suggested candidate biomarkers in the published literature, the purpose here is not to be comprehensive, but to give a general overview of some of the most frequently explored options for providing a level of comparison with RECIST.

**Table 4:** Reproducibility and factors impacting it of other imaging biomarkers.

Biomarker	Reproducibility			Factors impacting reproducibility
	ICC	Coefficient of Variation	Other	
Metabolic activity (^18^-FDG PET) *Semiquantitative*: SUV (SUV_max_, SUV_mean_; SUV_peak_), SUL (SUL_max_, SUL_mean_, SUL_peak_); MTV, TLG *Response criteria*: PERCIST (30)/EORTC (31)	SUV_max_ (4 observers) (22) - Pre-treatment: 0.93 - Post-treatment: 0.91 - Interval change: 0.94 - SUV_mean_ repeatability (32) - 0.91 (meta-analysis) SUV_peak_ - -31% to 30%	SUVmax (4 observers) (22) - Pre-treatment: 6.3% - Post-treatment: 18.4% - Interval change: 16.7%	Repeatability standard deviation (33) - SUV_max_: 1.01 - SUV_mean_: 0.28	*Technical factors*: Scanner calibration/injected activity calibration (34, 35) Incorrect decay correction (36) Tracer extravasation (37, 38) Residual activity in syringe (34) Synchronization of clocks (34) *Biological factors*: Blood glucose levels (38) Inflammation (34) Patient preparation (38) Injection-acquisition interval (39, 40) BMI/metabolic syndrome (41) Drug interaction/corticosteroids (38) *Physical factors:* Acquisition parameters/matrix size (34, 36) Reconstruction algorithm (39, 42, 43) Partial volume effect (44) Normalization factor for SUV (34, 45) Use of contrast agents (34) ROI/VOI definition (39, 42) Semiautomated/manual contouring (46) Movement artifacts/respiratory movements (34) Recovery effect/motion blur (47) Image noise (44, 48) Background activity/visual assessment (42, 49) Lesion size/location (50)
Vascularity (DCE MRI)	DCE-MRI ktrans - Intra-obs: 0.98 (51) DCE CT (arterial flow, blood volume, permeability) - Intra-obs: 0.72-0.89 - Inter-obs: 0.70-0.91 (52) DCE and DSC-MRI intersoftware reproducibility ICC 0.31 to 0.58 (53)	DCE MRI - model-free parameters (ex: AUC_60_, peak…): 12-24% - modelled parameters (ex: distribution volume, blood flow, mean transit time): 21-29% (54) DSC MRI normalised rCBVmax - repeatability: 50%, - reproducibility: 6% (55) DCE-CT (blood flow, blood volume, mean transit time, permeability) - within subject: 18% to 25%; DCE-MRI (Ktrans, k(ep), v(e)) - within subject 16% to 23% (56),		- Parameter extraction model (54) - Segmentation: 3D vs 2D regions of interest (52) - Software (53)
Cellularity (MR) ADC	ADC mean value - Intra-obs: 0.91 (57) – 0.99 (51) - Inter-obs: 0.92 (57) ROI segmentation method (Inter-obs) - Manual method: 0.69 - Semi-automated volumetric method: 0.96 (58)	Repeatability - ADC total = 4.8% (57), 7.1% (59) to 13.3% (60) Different post-processing platforms - 2.8% (59) Different sites - multicentric: 9% (61) - ice-water phantom: 1.6% (61) - breast fibroglandular tissue: 7.0% (61)	Repeatability (single centre) - ≤ ± 0.1x10^-3^ mm^2^/s (62)	- Field homogeneity gradient linearity (63) - QA procedure by trained operators assessing artifacts, fat suppression, and signal-to-noise ratio (57) - Segmentation: 2D vs. 3D, manual vs. semi-automatic (58) - Choice of measurement: mean/min/max/percentiles of ADC (64) - Lesion size (59)

SUV_max_ is measured as the maximum single voxel value of SUV, SUV_mean_ is the average value of SUV in all voxels above a threshold, SUV_peak_ (is the average value of SUV in a region of interest positioned so as to maximize the enclosed average.

SUV, standardized uptake value; SUL, lean body mass corrected SUV; MTV, metabolic tumour volume; TLG, total lesion glycolysis; PERCIST, PET Response Criteria in Solid Tumours; EORTC, European Organization for Research and Treatment of Cancer; wCV, within-subject coefficient of variation; BMI, body mass index; ROI, region of interest; VOI, volume of interest; ICC inter correlation coefficient; DCE dynamic contrast enhanced; DSC-MRI dynamic susceptibility contrast magnetic resonance imaging; ADC, apparent diffusion coefficient; QA quality assurance; 3D, three-dimensional; 2D, bi-dimensional; AUC_60_, area under the curve at 60s; rCBV, relative cerebral blood volume; Ktrans, transfer constant; k(ep), wash-out transfer constant; v(e), extracellular volume.

A first alternative to measuring a single size dimension as a response biomarker, would be to measure volume of a single or several lesions as an indicator of tumour bulk. This seems particularly important when lesions are irregular in shape, or when they change orientation and are therefore not identically represented on standard axial follow-up scans. Volumetric response on first follow-up CT has been shown to better predict OS than RECIST response (65). Tumour volume response has been utilised in lung (66), cervical (67), and other solid malignancies (68). Despite a trend towards better intra- and inter-observer reproducibility, the routine use of volume has been hampered by the need for manual segmentation, which is user-dependent and time-consuming and does not improve the discrepancies linked to the choice of target lesions (24, 28). Aside from tumour bulk, metabolic activity of tumours through functional imaging (e.g. positron emission tomography - PET)) is highly predictive of response in lymphoma (69), lung cancer (70), and metastatic melanoma (71). Other radioligands are utilised for response or recurrence detection, e.g. ^18^F-fluoroestradiol (FES) in hormone-dependent breast cancer (72) and ^18^F- or ^68^Ga Prostate-Specific Membrane Antigen (PSMA) ligands in prostate cancer (73). Additionally, radiolabelled ligands of various metabolites and biologically active molecules can assess proliferation, hypoxia, angiogenesis, apoptosis and gene transfection (74).While parameters used for the quantification and measurement of tumour metabolism by PET are generally based on semi-quantitative assessments, these can be made relatively reproducible and harmonised throughout the world through standardised imaging protocols and dedicated initiatives promoted by the international scientific societies (75, 76), such as the accreditation program developed by the EANM Research Ltd. (EARL) (34, 77).

Other alternate imaging biomarkers include perfusion and diffusion imaging. As tumours are commonly characterised by neo-angiogenesis, perfusion and permeability derived from dynamic-contrast enhanced studies (e.g. with MR or CT) have been contenders for measuring early response (78), and vascularity can be quantified using most imaging techniques, such as MRI, CT, ultrasound and PET. The utility of biomarkers of vascularity has been demonstrated particularly where anti-angiogenic agents such as bevacizumab have been part of the therapeutic strategy (79). However, their quantitation, which depends on measuring or estimating an arterial input function, is susceptible to large potential variations (80), and the reproducibility of such data is often low, thus limiting their clinical utility (81). Another biomarker reflecting tissue cellularity, the apparent diffusion coefficient (ADC) from DW-MRI, has proven a robust quantitative measure with good repeatability and reproducibility across vendor platforms (82), and has the potential to detect therapeutic response earlier than size measurements. It is increasingly being introduced routinely into scanning protocols, as it does not require injection of an extrinsic contrast agent and is simple and fast to acquire and analyse. Increasing automation with artificial intelligence (AI) systems may aid the translation of biomarkers indicative of tumour characteristics other than bulk into routine clinical workflows. Unfortunately, tightened legal rules are slowing down the process of their adoption (83).

Although historically dependent on imaging, response assessment for malignancies may now also include liquid biopsies [quantification of circulating tumour cells or DNA (CTC, ctDNA)], as well as histological sampling. ctDNA shedding is influenced by the overall tumour burden (cells) and may thus inform the use of imaging in relation to likely tumour size (84), because ctDNA estimations require less workflow and infrastructure than repeated monitoring with imaging. Initial clinical evaluations showed that ctDNA detected response earlier than imaging-based assessment (85). The simplest clinical implementation of ctDNA may be in postoperative monitoring of disease recurrence (86) but even here reproducibility and standardisation issues remain limiting. In one study, ctDNA quantities based on measurement of some target genes (e.g. TERT) were, on average, more than two-fold higher than those of other assays (e.g. ERV3) (87). In another, quantities of cell-free DNA for the different isolation methods for detection of EGFR variants in NSCLC varied between medians of 1.6 ng/mL and 28.1 ng/mL (88). Moreover, concordance between tissue and plasma variant detection for leading platforms has been shown to range from 70% to 90% (89). Thus, ctDNA extraction/isolation methods (87, 88) may need to be standardised before routine clinical use.

Finally, histopathology may also be a method for tumour response evaluation. However, serial histological sampling is not routinely used for response assessment and has thus far shown agreement with imaging-based responses only in a few studies (90). Histopathological evaluation of response is performed usually after neoadjuvant therapy, when the organ is surgically resected. Qualitative or semi-quantitative histopathological evaluation also presents variable reproducibility according to organs, methods and published studies (91–94). Agreement between pathologists yielded kappa values ranging from 0.21 for extent in prostate cancer (92), to 0.49 for multiple well-trained observers in cervical cancer (93), 0.64 for a 5-point tumour regression grade in rectal cancer (90) and 0.83 for a central review in bladder cancer (91). As with macroscopic imaging, reader experience (94), and central review (92) improve reproducibility.

### Statement #4:

*Alternative biomarkers for tumour response yield reproducibility generally comparable to RECIST. Each technique has its sources of variability, and it is important to understand inherent variability and limitations of individual biomarkers. It is critical that imaging experts communicate their level of confidence in any chosen biomarker.*

## What Are Common RECIST Limitations?

### Challenging Organs: Bone

Bone metastases were considered unmeasurable in the initial RECIST initiative, because of the lack of sensitivity of existing techniques to bone marrow infiltration (4). On CT it is the bone’s osteolytic or osteosclerotic reaction to the presence of tumour, or its response to therapy (flare lesions) that is visualised rather than the tumour itself (95, 96). With the updated RECIST 1.1. version, bone metastases with soft tissue masses ≥10 mm are recognized as measurable target lesions (1). Nevertheless, bone lesions without soft tissue involvement, whether lytic, mixed or sclerotic, remain unmeasurable by RECIST. Since the early 1990s, bone marrow MRI has been shown to be superior to bone scintigraphy and CT for the assessment of bone metastatic disease. Bone marrow replacement by neoplastic foci is detected and quantified on T1-weighted and fat-suppressed T2-weighted MRI sequences (97, 98), more recently complemented with diffusion-weighted imaging (DWI) sequences (99, 100). However, to date, RECIST 1.1 has not validated quantitative bone MRI for tumour response assessment. Positron Emission Tomography Response Criteria in Solid Tumours (PERCIST), introduced in 2009 (30, 101), enables response to be measured in ^18^F-fluorodeoxyglucose (^18^F-FDG) avid bone metastatic lesions based on their metabolic activity in the absence of any obvious anatomic changes. Finally, PSMA-PET appears promising for identifying bone marrow invasion due to prostate cancer, regardless of the impact on the bone mineral content (102, 103).

### Challenging Diseases: GIST and mCRC

As RECIST is not organ-specific, it might not capture the key parameters that are associated with survival outcomes in certain cancer types, and under certain types of treatment. In gastrointestinal malignancies, the hepatic tumour burden and its response commonly outperform other sites of metastatic disease for survival prediction. A study in metastatic colorectal cancer (mCRC) showed that the depth and uniformity of response in liver metastases represented a highly useful and clinically relevant indicator for therapy monitoring (104). Organ-specific response patterns may also occur under immunotherapy possibly due to varying immune microenvironments in organs or the lymphatic system (105–107). Thus, choice of target lesions would largely impact the response observed according to the organ, as well as the predictive ability of RECIST. In this case also, reader experience and knowledge of the disease is crucial for proper target lesion selection.

**Figure 2:**
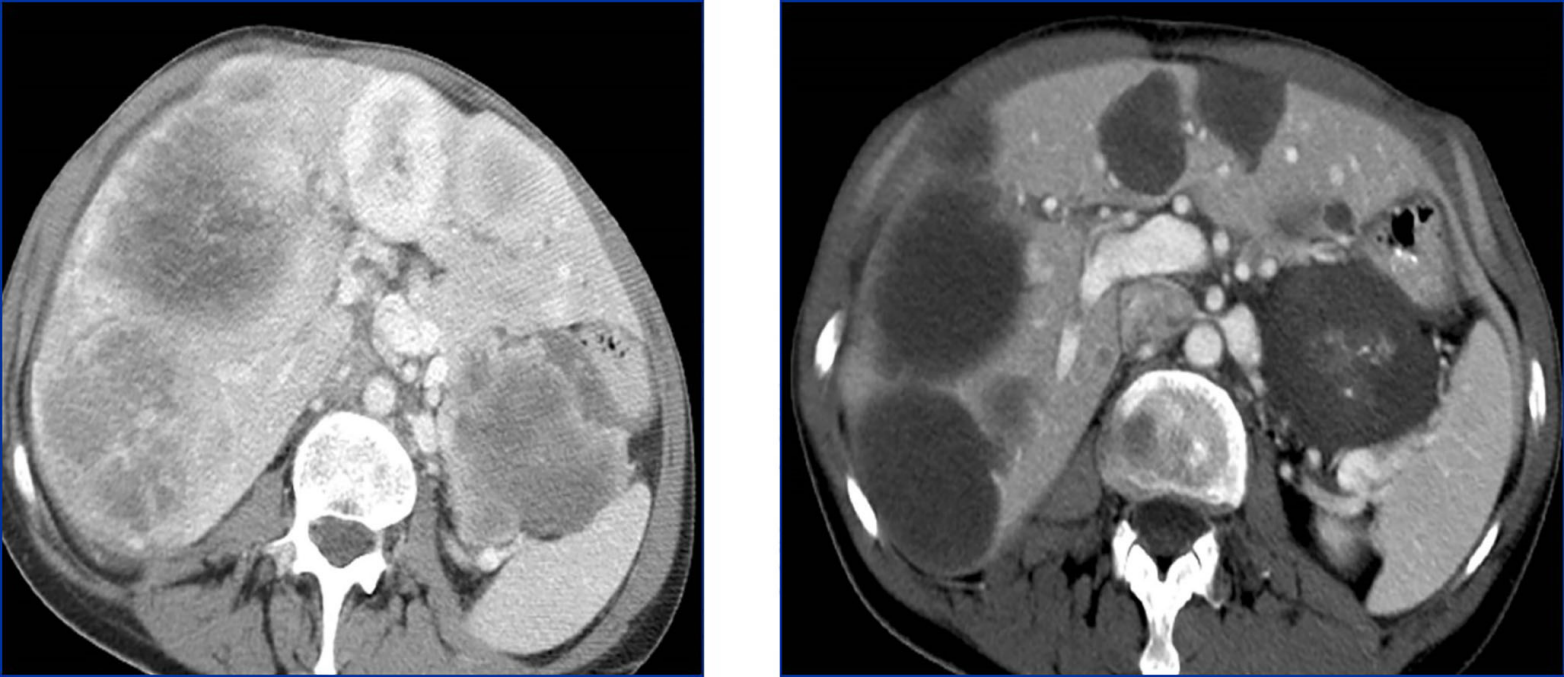
Response unrelated to tumour size in a 66 yo patient treated with imatinib for a gastrointestinal stromal tumour (GIST). Compared to the baseline image (left), after treatment (right) the tumour shows a dramatic decrease in density rather than in size.

Response to therapy in patients with advanced GIST was drastically improved by the introduction of imatinib, a tyrosine-kinase inhibitor. Imatinib treatment has been shown to induce necrosis with a marked decrease in vascularity of GIST lesions, resulting in a decrease in CT density often before any significant decrease in size is seen, thus leading to underestimation of the initial tumour response (108, 109) (**Figure 2**). A paradoxical increase in volume is occasionally observed, simulating progression (110). Choi et al. therefore proposed adapted criteria for GIST, combining changes in tumour density on contrast-enhanced CT expressed in Hounsfield units (HU) and/or size to determine tumour response (109): PR is defined as a decrease of ≥10% in the SOD or a decrease of ≥ 15% in tumour density of target lesions, whereas PD is defined as a ≥ 10% increase in size and not meeting the PR criteria by tumour density. PD may also occur if new intra-tumoural nodules are present or existing intra-tumoural nodules show an increase in size, factors which are not catered for in RECIST. In patients treated with imatinib, Choi criteria showed a significantly better correlation with survival rates than RECIST (111).

### Challenging Treatments: Focal Therapies

Treatment of tumour lesions with ablative therapies, such as radiofrequency ablation, microwave ablation or cryoablation, results in a larger defect than the original lesion and such treated lesions are not considered measurable unless there is progression at this site (1), such as the development of a new measurable nodule within the ablation defect. Distinguishing normal post-ablation changes from residual disease and recurrence can be challenging (112).

Intravascular therapies are also a challenge for the use of RECIST. Trans-arterial radioembolization (TARE) induces inflammatory changes with a generally delayed morphologic response (112). A reduction of ^18^F-FDG uptake on early PET-CT has been found to be helpful in predicting further outcome of these patients (113). As a consequence, both TARE and intra-arterial therapies such as trans-arterial chemoembolization (TACE) in hepatocarcinoma require modified RECIST (mRECIST) criteria derived from arterial and portal venous enhancement phases of CT or MRI (114), and which take into account both lesion size and vascularity.

High-intensity focused ultrasound (HIFU), under the guidance of ultrasound or MRI, has also been used as a non-invasive technique for tissue ablation in prostate cancer and more recently in recurrent gynecological malignancy (115). The use of HIFU for hepatic tumour lesions is still in the exploratory stage. As for other ablative therapies and for similar reasons (116), RECIST 1.1 appears to be unsuitable for local response evaluation following HIFU applied to liver lesions.

Finally, tumour lesions in a previously irradiated area (via CyberKnife, stereotactic radiotherapy or traditional fractionated radiation therapy) are not considered measurable (1) and must be excluded from RECIST evaluation due to the inflammatory or fibrotic changes that may be observed, thus making evaluation of size unreliable.

### Statement #5

*There are several scenarios in which RECIST criteria fail to evaluate treatment-induced changes correctly. Informed appreciation that RECIST criteria are not applicable to all tumour sites and situations is thus crucial for proper interpretation and again dependent on reader experience.*

## When Is RECIST Response Assessment Misleading?

### Pseudo-Progression

**Figure 3:**
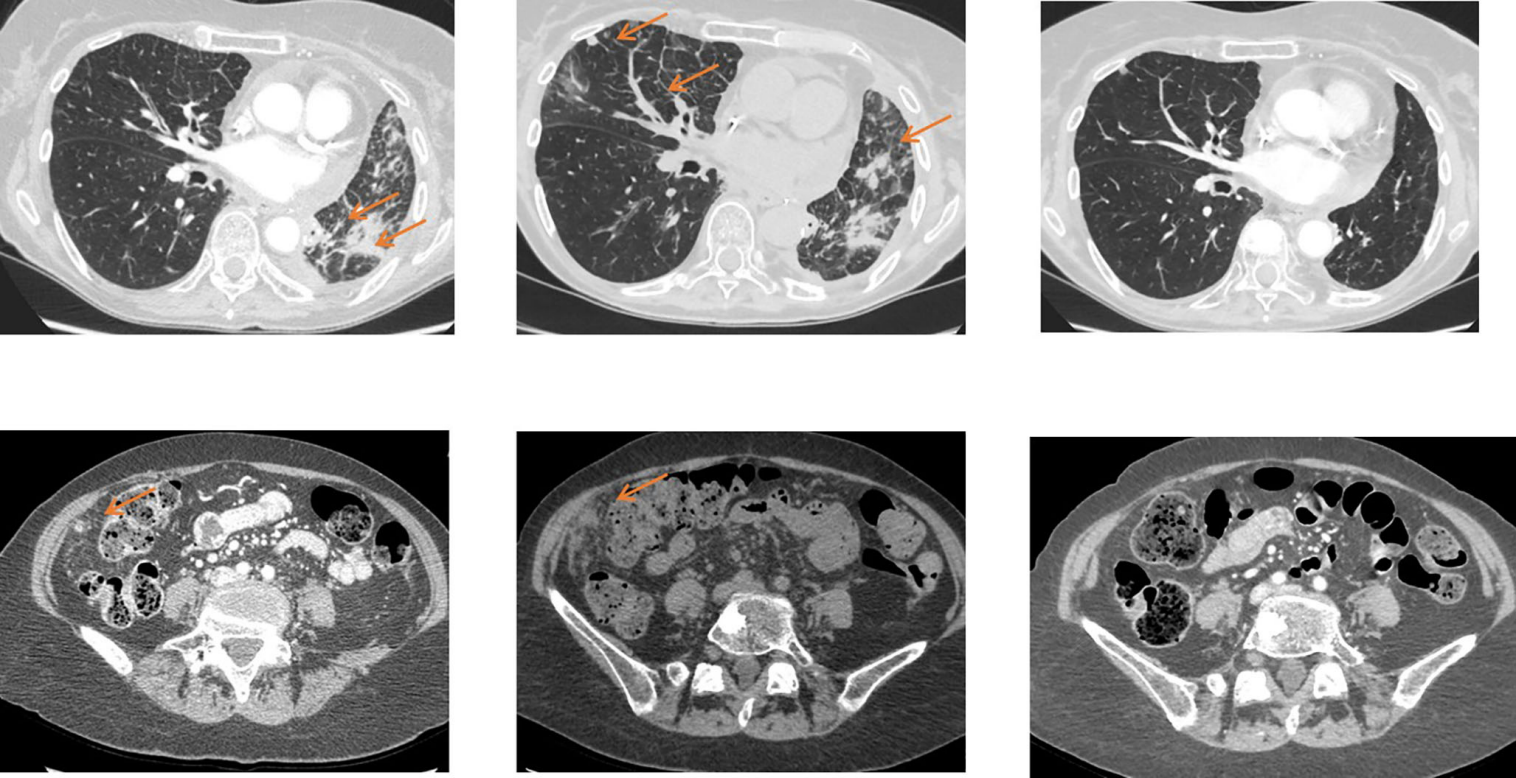
Pseudoprogression on immunotherapy in a 56 yo patient with metastatic non-small cell lung cancer. The baseline image (left) shows lung and peritoneal nodules (arrows). After 4 wks of antiPDL1 therapy (middle), CT shows an increase in previous lesions and the appearance of new lung nodules. Disease was considered immune unconfirmed progressive disease. Six weeks later (right) a dramatic response in all previous lesions was seen classifying the patients as a complete responder and endorsing an earlier diagnosis of pseudoprogression.

During immunotherapy, RECIST may describe progression that can be misleading and is thus classified as “pseudo-progression”. In fact, in around 5 to 10% of patients with metastatic disease treated with check-point inhibitors, an initial increase of tumour burden has been observed, followed by actual response or long-term stabilisation of disease (117–119). This phenomenon relates to the mechanism of action of immunotherapy, which stimulates the immune response and initially induces inflammation and tumour swelling, thus delaying visible tumour shrinkage. For this reason, adaptations of RECIST criteria for assessing treatment response to immunotherapy (iRECIST) have been developed. The first ascertainment of progression by iRECIST is considered as “immune unconfirmed progressive disease”(or iUPD), and requires, if possible, a subsequent evaluation 4 to 8 weeks later in order to confirm true progression (120) (**Figure 3**).

### Mixed Response/Progression

In some patients, the tumour bulk does not respond homogeneously, with some lesions increasing and others decreasing. Mixed or heterogeneous response is defined as an increase in size of some tumour lesions and decrease of others in the same patient during treatment. This lesion-specific response has been attributed to the emergence of drug-resistant clones and indicates that tumour heterogeneity is likely causing treatment failure (121, 122). Mixed response has the same incidence in patients treated with targeted cancer agents and those undergoing chemotherapy alone or even combined with targeted agents (12, 28).

**Figure 4:**
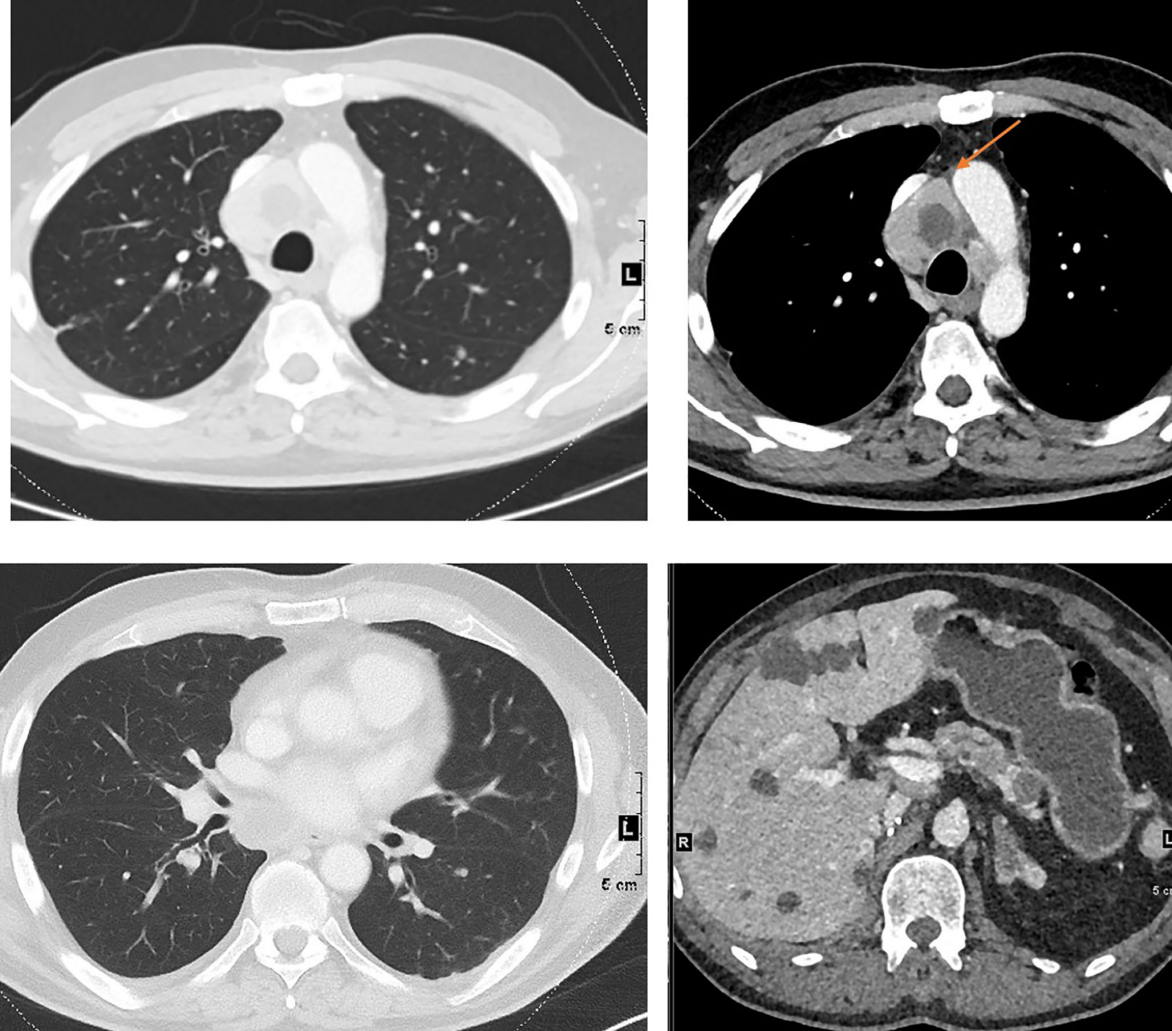
Mixed response to treatment in the same patient illustrated in **Figure 1**. Eight weeks after targeted therapy lung, adrenal and pancreatic metastases decreased, whereas one mediastinal lymph node (top right, arrow) increased.

Since RECIST records overall patient response rather than individual lesion response, the choice of target lesions critically affects the objective assessment of overall patient response in patients with mixed response in individual lesions (**Figure 4**) (12). As lesions escaping treatment control will weigh negatively on patient prognosis (123), their presence should be annotated in order to offer the best alternative treatment for the patient.

Lesion cavitation, necrosis and residual non-viable masses represent other forms of response than decrease in size and may complicate RECIST assessment (124). Tumour necrosis with cavitation is present in approximately 14-24% of NSCLC patients undergoing anti-angiogenic drug therapy (125–127). When cavitation is present, lesion size may not change significantly and RECIST may therefore under-estimate the effect of therapy. Conversely, cavitation also risks missing progression if there is tumour regrowth inside the cavity. While alternative criteria have been proposed in such cases, e.g. subtracting the longest cavitation diameter from the largest lesion diameter (such as Crabb criteria) (126), these are not commonly used.

When residual tissue is present after therapy, evaluation with RECIST criteria is subject to pitfalls. First, an asymmetric shrinkage of the tumour may result in a similar longest diameter and consequent stable disease (SD) rating not reflecting the real response to treatment (**Figure 2**). Second, it may be difficult to distinguish between viable tumour and fibrosis. In such cases, best response assessment, an important endpoint in phase 2 studies (partial vs. complete response; PR vs. CR) may be affected (126). According to RECIST guidelines, in equivocal cases, residual lesions should be evaluated by either biopsy or PET(-CT) (**Figure 2**). This may well then allow upgrading PR to CR. However, false positive PET findings are not uncommon (128). Alternatively, other advanced imaging tests, such as DWI-MRI or perfusion imaging (e.g. from MR or CT) could be used.

### Statement #6

*Some patterns of response/progression cannot be correctly documented by RECIST. These require specialist reader experience and communication with oncologists to determine appropriate evaluation approaches and/or therapeutic options.*

## Should (Could) RECIST be Automated?

The core assumption of RECIST is that a single diameter on the cross-sectional imaging slice presenting the largest cross-section of a given lesion (or sum thereof) is a surrogate for tumour burden. This assumes that lesions are grossly spherical and that their size represents their overall activity. To streamline the determination of this single diameter and make it less subject to possible human-induced variability, semi- or fully-automated 2D or even 3D segmentation techniques can be applied to target lesions, which can also be semi- or fully-automatically tracked between scans acquired at different time points (129–134). The 2D or 3D mask resulting from the segmentation process then readily permits the automated and accurate extraction of the largest diameter from the segmented lesion. With 3D segmentation, the full volume of a target lesion can be provided alongside an automatically extracted largest diameter, which may not be oriented in the 2D plane of the source images in a broader RECIST interpretation, together with any other geometric metric of relevance. Using the largest 3D diameter would allow RECIST to be used beyond 2D constraints, and can account for non-orthogonal motion of target lesions between scans at different time-points. While segmentation and tracking can now plausibly be fully automated, especially with newer approaches using machine learning, and such capabilities are already implemented in several commercially available clinical systems, some challenges remain with key RECIST operations, such as the proper selection of target lesions and dealing with new or disappeared lesions. These are currently still best addressed or verified with a human (e.g. a radiologist) in the loop (20, 135).

### Statement #7

*Though automation is desirable to streamline the process and potentially reduce the variability of results within the RECIST paradigm, remaining technical challenges must be overcome to ensure proper repeatability, and human adjudication is still required.*

## RECIST in Novel Drug Development

RECIST measurements play a pivotal role in the development of novel oncological drugs (136). In most registered randomised controlled trials (RCTs), studies are powered to meet primary endpoints such as OS/PFS, which determines the number of patients recruited. A study of RCTs between 2006 and 2015 looking for evidence of clinical efficacy of novel oncology drugs in order to gain US Food and Drug Administration (FDA) approvals had PFS as primary endpoint in 28 out of 42 RCTs (66%), and OS in 14 (33%). In 2012, 12 novel anticancer drugs were approved by the FDA; only three drugs showed improvement of overall survival (137). Similarly, a study of drugs approved by the European Medicine Agency (EMA) between 2009 and 2013 also showed that only 18 of 68 (26%) novel drug uses were supported by OS data, whereas PFS was used in 31 (46%) (138). In the vast majority of trials, PFS is determined using the RECIST1.1 framework, or iRECIST for immune-oncology trials. It is acknowledged however, that in some disease types other criteria are used: e.g. Lugano criteria for ^18^F-FDG PET/CT or RECIL in lymphoma (139, 140) and RANO criteria for brain tumours (141, 142). The fact that PFS can predict OS outcome in large patients cohorts with commonly occurring cancers, reinforces the use of RECIST criteria in clinical trials (143). Moreover, rapid progress in drug development will make the reliance on OS as endpoint for novel drugs in oncology increasingly challenging because treatment options on progression on trial, including in-trial cross-over, are increasing.

### Statement #8

*Although the RECIST framework might not be perfect, the scientific basis for the anticancer drugs that have been approved using a RECIST-based surrogate endpoint remains valid.*

## RECIST: Only as Good as its Users?

RECIST criteria were developed for clinical trials and thresholds chosen to produce a comparable classification of patients, taking into account tumour measurement variability. These criteria are widely used in clinical trials and accepted by regulatory agencies. Despite some limitations, the scientific basis for the anticancer drugs that have been approved using a RECIST-based surrogate endpoint remains valid. Reader experience, choice of target lesions and detection of new lesions impact RECIST reproducibility, which necessitates adequate training of radiologists using these criteria. Automation is not currently sufficiently reliable to replace human experience. Unfortunately, some organ-, disease- or drug-specific patterns of response/progression cannot be correctly documented by RECIST.

This expert statement includes that RECIST remains a tool for radiologists that needs to be used with discrimination and good understanding of its purpose and limitations. Training of radiologists is essential to improve its application and reproducibility. RECIST conclusions should not go against common (or informed) sense. Furthermore, RECIST criteria have the advantage of simplicity, availability, cost-effectiveness, and intuitiveness. Overall, therefore, RECIST provides a common language between oncologists and imaging experts (e.g. radiologists), provided there is full understanding of how measurements are made, what they represent, and their inherent limitations.

## Data Availability Statement

The original contributions presented in the study are included in the article/supplementary material, further inquiries can be directed to the corresponding author.

## Author Contributions

All authors contributed to conception and design. CC and LF wrote the first draft of the manuscript. All authors contributed to manuscript revision, read, and approved the submitted version.

## Conflict of Interest

The authors declare that the research was conducted in the absence of any commercial or financial relationships that could be construed as a potential conflict of interest.

## Publisher’s Note

All claims expressed in this article are solely those of the authors and do not necessarily represent those of their affiliated organizations, or those of the publisher, the editors and the reviewers. Any product that may be evaluated in this article, or claim that may be made by its manufacturer, is not guaranteed or endorsed by the publisher.
